# The REFANI-S study protocol: a non-randomised cluster controlled trial to assess the role of an unconditional cash transfer, a non-food item kit, and free piped water in reducing the risk of acute malnutrition among children aged 6–59 months living in camps for internally displaced persons in the Afgooye corridor, Somalia

**DOI:** 10.1186/s12889-017-4550-y

**Published:** 2017-07-06

**Authors:** Mohamed Jelle, Carlos S. Grijalva-Eternod, Hassan Haghparast-Bidgoli, Sarah King, Cassy L. Cox, Jolene Skordis-Worrall, Joanna Morrison, Timothy Colbourn, Edward Fottrell, Andrew J. Seal

**Affiliations:** 1 UCL Institute for Global Health, WC1N 1EH, London, UK; 2 Concern Worldwide Somalia, Nairobi Office, Nairobi, Kenya

**Keywords:** Acute malnutrition, Wasting, Cash transfer, Somalia, Study protocol, Internally displaced persons, Food security, Children

## Abstract

**Background:**

The prevalence of acute malnutrition is often high in emergency-affected populations and is associated with elevated mortality risk and long-term health consequences. Increasingly, cash transfer programmes (CTP) are used instead of direct food aid as a nutritional intervention, but there is sparse evidence on their nutritional impact. We aim to understand whether CTP reduces acute malnutrition and its known risk factors.

**Methods/design:**

A non-randomised, cluster-controlled trial will assess the impact of an unconditional cash transfer of US$84 per month for 5 months, a single non-food items kit, and free piped water on the risk of acute malnutrition in children, aged 6–59 months. The study will take place in camps for internally displaced persons (IDP) in peri-urban Mogadishu, Somalia.

A cluster will consist of one IDP camp and 10 camps will be allocated to receive the intervention based on vulnerability targeting criteria. The control camps will then be selected from the same geographical area. Needs assessment data indicates small differences in vulnerability between camps.

In each trial arm, 120 households will be randomly sampled and two detailed household surveys will be implemented at baseline and 3 months after the initiation of the cash transfer. The survey questionnaire will cover risk factors for malnutrition including household expenditure, assets, food security, diet diversity, coping strategies, morbidity, WASH, and access to health care. A community surveillance system will collect monthly mid-upper arm circumference measurements from all children aged 6–59 months in the study clusters to assess the incidence of acute malnutrition over the duration of the intervention. Process evaluation data will be compiled from routine quantitative programme data and primary qualitative data collected using key informant interviews and focus group discussions.

The UK Department for International Development will provide funding for this study. The European Civil Protection and Humanitarian Aid Operations will fund the intervention. Concern Worldwide will implement the intervention as part of their humanitarian programming.

**Discussion:**

This non-randomised cluster controlled trial will provide needed evidence on the role of unconditional CTP in reducing the risk of acute malnutrition among IDP in this context.

**Trial registration:**

ISRCTN29521514. Registered 19 January 2016.

## Background

Acute malnutrition in childhood is a serious global health concern affecting an estimated 50 million children aged <5 years in low- and middle-income countries [1, 2]. Acute malnutrition is a leading cause of childhood mortality, accounting for 11.5% of total deaths, and contributes significantly to the overall disease burden [3]. The prevalence and severity of acute malnutrition is usually greater in populations affected by emergencies- such as in natural disasters or conflicts [4].

**Fig. 1:**
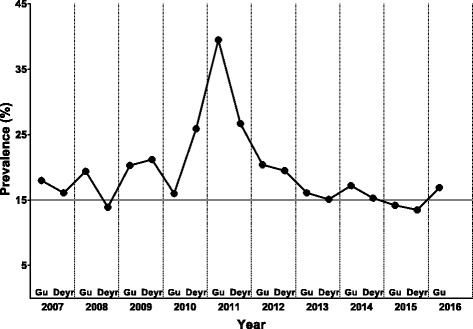
Trends in Global Acute Malnutrition (GAM) in the south-central region of Somalia. Adapted from reference [6]

Because of a prolonged state of instability and conflict, coupled with natural disasters, Somalia has one of the highest global prevalences of child acute malnutrition [5]; with the south-central region consistently exceeding the critical prevalence threshold of 15% (Fig. 1) [6]. The state of conflict has disrupted not only regional agriculture and trade, but also humanitarian access, forcing displacement and increasing mortality [7]. Furthermore, trade disruption has exacerbated food insecurity due to increased food prices, especially in urban areas [7]. An estimated 304,700 children aged <5 years were acutely malnourished in Somalia in early 2016, of which 58,300 were severely malnourished [6]. The group most affected by food insecurity and acute malnutrition are internally displaced persons (IDP), who often live in camps.

There are various nutritional interventions commonly used for the management of acute malnutrition [8]. Among these, cash transfer programmes (CTP) have recently gained popularity compared to conventional food-based interventions [9]. CTP may involve a conditional or unconditional cash or voucher transfer to beneficiaries, with the aim of improving their ability to acquire food and/or other needs. While food security and nutrition are objectives often stated for CTP, they frequently have multi-sectoral objectives and may also be intended to enable livelihood investments [10, 11]. Furthermore, CTP are perceived to reduce the cost of nutritional and related interventions, improve beneficiary satisfaction, and have a positive impact on local economies [11]. CTP were first implemented at scale in Somalia in 2011 to respond to the famine crisis. CTP were made possible by the existence of functioning markets and were seen as an essential approach given the lack of humanitarian access to parts of South Central region that made the provision of food aid impossible [12]. Nonetheless, the suitability and nutritional impact of CTP needs further evaluation in order to measure their impact on acute malnutrition in emergencies and understand the mechanisms through which they may act.

## Methods/design

### Objectives

To conduct a non-randomised cluster-controlled trial to assess the impact of an unconditional CTP on acute malnutrition and its associated risk factors in children aged 6–59 months, living in IDP camps in the Afgooye Corridor, a region close to Mogadishu, Somalia.

### Hypotheses

We have two hypotheses. 1. Unconditional cash transfers reduce the risk of developing acute malnutrition in children aged 6–59 months. 2. Unconditional cash transfers reduce known risk factors for acute malnutrition in IDP camps located in a peri-urban area of Mogadishu, Somalia.

### Study setting and population

The study will be conducted in IDP camps located in Weydow area, Deyniile district, Mogadishu. Deyniile and Dharkenley, two of the 17 districts of Banaadir region, host the majority of IDP in Mogadishu, who are primarily from marginalised tribes or minority groups [13]. Concern Worldwide (hereafter Concern) has been implementing multi-sector development and humanitarian assistance programmes in this setting.

The IDP camps in Weydow area are privately run, spontaneous settlements that are often overcrowded, lack basic sanitation and health services, and face recurrent evictions. Morbidity (diarrhoea, pneumonia, and fever) estimates in these camps are high and may be a major driver for high global acute malnutrition (GAM) estimates [6]. During the wet seasons (Gu and Deyr, Apr-Jun and Oct-Dec, respectively), morbidity estimates are particularly high, mainly due to diarrhoea [6].

Most of the IDP were previously agro-pastoralists and riverine farmers that lived in the Bay, Bakool, and Shabelle regions [13]. Currently, their primary livelihoods sources are casual labour, petty trading, and humanitarian assistance received from local and international humanitarian organisations [6].

### Intervention

The intervention will comprise a monthly unconditional CTP of US$ 84.00/month for 5 months, a once-only distribution of a non-food items (NFI) kit, and the provision of piped water through tap stands.

**Fig. 2:**
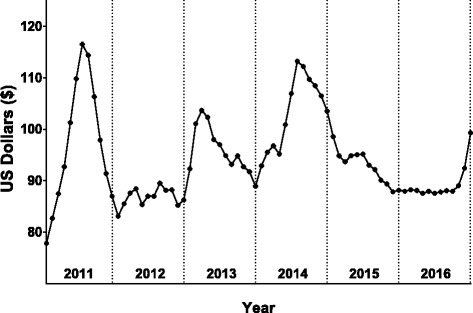
The cost of a Minimum Expenditure Basket (MEB) in the Banaadir region, 2011–2016. Data obtained and adapted from the FSNAU website (http://www.fsnau.org/sectors/markets). The MEB cost is calculated based on a minimum set of basic food items such as sorghum, vegetable oil and sugar, that will supply 2100 kcal/person/day for a household of 6–7 members, and non-food items such as such as water, kerosene, firewood, soap, and cereal milling costs

The monthly cash amount was based on the cost of the Minimum Expenditure Basket (MEB) developed by the FAO’s Food security and Nutrition Analysis Unit (FSNAU). The MEB represents a minimum set of basic food items such as sorghum, vegetable oil and sugar, comprising 2100 kcal/person/day basic energy requirement for a household of 6–7 members, and non-food items such as such as water, kerosene, firewood, soap and cereal grinding costs. As a reference, Fig. 2 shows the historical cost of MEB in the Banaadir region from January 2011 to December 2016.

Upon registration, a household female representative will receive a mobile phone SIM card with a unique number, through which they will receive the transfer via a mobile money transfer company. The intervention will target women as the household cash recipient, on the assumption that their spending is more likely to benefit their children [14].

The NFI kit will comprise one plastic sheet, two mosquito nets, one blanket, one sleeping mat, one kitchen set, one bar of soap, two collapsible jerry cans, and one set of sanitary pads.

Concern will also continue to provide support and equipment to the local Maternal and Child Health and Nutrition (MCHN) centres and Outpatient Therapeutic Programmes (OTP), where malnourished children and pregnant and lactating mothers of the study area will be referred for treatment. Improved pit latrines have been provided by Concern and other NGOs in most of the camps included in the study.

### Study design

The study design is a non-randomised cluster controlled trial. Concern will implement a routine needs assessment exercise to identify vulnerable beneficiaries and on that basis select the camps that will receive the intervention. All households in the selected camps will be registered for the intervention. A non-randomised design was chosen as the best available approach in this setting because the intervention will be allocated based on vulnerability criteria.

The cluster unit in the trial will be an IDP camp and the intervention arm will include 10 IDP camps selected to receive the intervention. The reason that only 10 camps can be selected to receive the intervention is the limit in the donor funding that is available to provide services in this area. The control arm will include another 10 IDP camps, located adjacent to the intervention camps that will not receive the intervention. Routine programme data suggests small differences in vulnerability between camps. Households in both arms will benefit from the services provided by Concern, such as care from OTP and MCHN centres.

### Study components

Data collection will consist of three main components:

1. *Household surveys of malnutrition risk factors*. To evaluate the impact of the intervention on known malnutrition risk factors two detailed household surveys will be implemented, the baseline survey will be undertaken before the intervention begins and the endline one 3–4 months after the start of the intervention.
  Based on our working assumption that insecurity in the field will constrain the possibility of following-up the baseline sample, the surveys will select independent samples of households from the study clusters at the two periods. If security conditions are worse than anticipated and do not allow sampling within a sufficient number of clusters, then we will collect data from a single control and intervention cluster and the data will be analysed as a before and after comparative cross-sectional study. Furthermore, if security conditions do not allow us to undertake repeated surveys in the same clusters, then we will undertake a single survey at the end of the intervention, and the data will be analysed as a cross-sectional study. However, if field conditions are better than anticipated, we will follow-up the baseline sample and the data will be analysed as a longitudinal cohort.
  The surveys will collect data at the household level and from all the children aged <5 years and their mothers/carers living in the selected households. The data collected in these surveys will comprise data on household demographics, household expenditure and income, assets, food security, diet diversity, coping strategies, morbidity, WASH, access to health care, and infant and young child feeding practices. The surveys will also collect anthropometric data from children to describe their nutritional status. However, this anthropometric data will not be used to assess the impact of the intervention due to sample size limitations (see below).
2. *A community surveillance system* will be set up to collect anthropometric data that will be used to evaluate whether the intervention reduces the risk of developing acute malnutrition. In this surveillance system, community health workers (CHW) will collect monthly mid-upper arm circumference (MUAC) measurements to detect the incidence of acute malnutrition in an exhaustive sample of children aged 6–59 months from both intervention and control clusters. Those identified as malnourished will be referred to nutrition centres for treatment.
3. *A process evaluation* will be conducted in order to better understand: the context in which the intervention was implemented; document how the intervention was implemented compared to how it was planned; explore the mechanism through which the intervention worked or failed to work, and investigate any unexpected outcomes. To achieve these objectives we will compile and analyse routine quantitative programme monitoring data and local health facility data on routine admissions and any disease outbreaks. We will also conduct qualitative data collection on IDP perceptions of malnutrition, risk factors, and the impact of cash transfers. Data will be collected using key informant interviews with community leaders, health staff and mothers and fathers, and focus group discussions with separate male and female groups of internally displaced people. These will explore how and why the interventions were effective, or not effective, in reducing child acute malnutrition.

### Sample size calculation

Sample size calculations were performed using Stata (StataCorp. 2015. Release 14. College Station, TX: StataCorp LP). For assessing the change in malnutrition risk factors using survey methods, the sample size was estimated to be able to detect a difference of 0.6 units in the mean individual dietary diversity score (IDDS). A positive change of about 2 units in the household diet diversity score was observed in response to a social CTP in Malawi [15]; but other studies had lower changes [16, 17]. There is little data on IDDS change following cash transfers so we decided to power the study to detect a conservative change, assuming a baseline IDDS of 3.0 with a SD of 1.5, with power of 80%, an alpha risk at 0.05, and an intra-cluster correlation coefficient of 0.01. The resulting required sample size was 120 children per arm. Based on previous nutrition survey data we assumed an average of 1.2 children, aged 6–59 month per household, which implies that 200 households will need to be sampled during each data collection period. Based on previous survey experience, we added 20% to allow for non-response leading to a required total sample of 240 households.

To assess malnutrition incidence using the community surveillance system and detect a hazard ratio of 0.5, we assumed, based on operational guidance for caseload estimations, that the proportion of the population under surveillance that would develop GAM during 6 months was 7%, and there would be 20% loss to follow up. Power was set at 80% and the alpha risk at 0.05. This resulted in a total sample size of 1167 children, which equated to a rounded sample of 600 participants per arm. Because data will be obtained from an exhaustive sample of children in both control and intervention clusters, we expect to have a greater sample.

### Data collection

Quantitative data will be collected using questionnaires translated into the local Somali language, and addressed to the primary carer of the child included in the study. A two-week training will be implemented for enumerators and supervisors prior to survey implementation. During this training we will pilot questionnaires. Survey data will be collected using mobile devices and community surveillance data will be collected on paper.

The study and data collection timelines are outlined in Table 1. Baseline and endline surveys will take place in March and June 2016, respectively. The community surveillance system will start data collection in March 2016 and will continue for about 1 year.

**Table 1:** REFANI-S intervention and data collection timeline

Study component	Month						
	Feb	Mar	Apr	May	Jun	Jul	Aug
Intervention		x	x	x	x	x	
Community surveillance system	x	x	x	x	x	x	x
Malnutrition risk factor surveys	x				x		

Four teams, each with two enumerators and led by two supervisors, will undertake data collection for the surveys. The qualitative data collection will be undertaken by one team with a supervisor. For the surveillance system, a total of 16 CHW will collect data, grouped into 4 teams of 4 members, each team with one supervisor. A field coordinator and a study coordinator will supervise all field-teams. All the study team except the study coordinator will be local staff and will be based in the Mogadishu area. Qualitative data will be collected by a trained team in the local Somali language.

### Outcome measures

The primary outcome measures are (1) incidence of acute malnutrition in children aged 6–59 months, as defined as a MUAC <12.5 cm and/or the presence of bilateral pitting oedema; and (2) mean individual diet diversity scores values of children aged 6–59 months.

The secondary outcome measures are (1) prevalence of GAM, as defined as a weight-for-length/height z-score < −2 (WHO 2006 Growth Standards) and/or bilateral pitting oedema in children aged 6–59 months; (2) mean weight-for-length/height values in children aged 6–59 months; (3) mean 30-day household expenditure; (4) mean household dietary diversity score, obtained using 24-h recall data; (5) mean household food insecurity access scale score, obtained using one-month recall data; (6) mean coping strategies index values, obtained using a 7-day recall data; (7) mean maternal MUAC; (8) mean maternal BMI.

### Anthropometric measurements

Body weight will be measured to the nearest 0.1 kg using a digital scale (Seca 874, Seca). Children will be weighed without clothes, and those unable to stand will be weighed with the caregiver, using the mother/child function of the scale. Women will be asked to remove all accessories, shoes and excess clothing before being weighed. Height (or recumbent length or height for children aged <24 months or measuring <87 cm) will be measured to the nearest 0.1 cm using a portable stadiometer (ShorrBoard, Shorr Production). MUAC will be measured on the left arm using an insertion tape to the nearest 0.1 cm. Bilateral pitting oedema on the dorsum of the foot will be assessed by applying medium pressure on both feet with thumbs for 3 s and determining the presence/absence of an indentation.

### Programme monitoring data

Routine programme monitoring and health facility admissions data will be collated and analysed using Excel 2013 to assess the implementation of the intervention and detect changes in the health and nutrition situation in the area. We will monitor and record the provision of relief interventions by other NGOs and any significant developments in security, economic situation, or infrastructure that may influence the health and nutrition situation of the IDP camp residents.

### Qualitative data

The study team will collect qualitative data regarding household members’ perceptions of malnutrition, its risk factors, and the perceived impact of cash transfers. The qualitative data collection will seek to: (1) provide context data to aid interpretation of the findings from the cross sectional surveys; and (2) provide case-narratives to better understand the role of cash transfers in the household economy and their potential role in reducing the risk of malnutrition. Collection of qualitative data will involve a purposively selected sample of cash receiving and non-cash receiving households. Data will be collected using Interviews, focus group discussions, and observation notes. FGD and interviews will be recorded using handheld tape recorders, transcribed in Somali, translated into English and analysed using the framework method for thematic analysis [18]. Analysis will be conducted using NVivo qualitative data analysis Software (QSR International Pty Ltd. 2012. Version 11).

### Data quality control

Enumerators will be trained in anthropometric measurements and standardisation tests will be undertaken to assess the precision and accuracy of their measurements [19]. Training of field staff on the use of questionnaires will be undertaken through classroom-based lessons, role-play, and piloting.

During field data collection, plausibility checks will be run daily on each team’s anthropometric data to assess the likelihood of data errors, using the Emergency Nutrition Assessment software [20]. Digital scales will be checked for accuracy daily using a standard known weight. Training of enumerators will ensure complete understanding of the questions. Qualitative fieldworkers will be recruited based on their skills and experience in collecting qualitative data and will be trained and supervised by an experienced qualitative researcher.

### Data analyses

Data analysis will be undertaken using Stata. Prevalence estimates at baseline and endline, will be computed using the *svy* commands which allows for the clustering of values. We will test for endline minus baseline difference-in-differences between the study arms using the data collected in the malnutrition risk factor surveys. Analysis of the incidence of acute malnutrition in the trial arms will be performed using Cox Proportional Hazards analysis. All results will be reported according to STROBE recommendations for observational studies [21].

### Study management

The study will be jointly managed by Concern and University College London (UCL). Concern will recruit staff and provide financial and logistical support for the field teams. UCL will provide the technical lead for the study design and implementation. Concern will implement the intervention.

### Study consortium

This study in Somalia is one of three studies that form the work of the Research on Food Assistance for Nutritional Impact (REFANI) Consortium; the other two have been implemented in Niger and Pakistan [22, 23]. The REFANI Consortium is comprised of Action Against Hunger (ACF), Concern, Emergency Nutrition Network and UCL.

## Discussion

This study began in March 2016 but due to field-based challenges, some modifications from the study protocol have occurred. Initiation of the unconditional CTP was delayed from March to May. Early preliminary data analysis of the surveillance system data also suggested that there was excess under-5 mortality in the study population and this prompted us to include Verbal Autopsy interviews, a method that will enable identification of the probable causes of death [24, 25].

Formative work has been undertaken and further qualitative work will be undertaken by the same team that did the quantitative data collection instead of a separate team as originally planned, because of the challenges of recruiting technical personnel with qualitative data collection experience that are willing to work in the field sites in Somalia. The staff involved have limited experience of qualitative data collection so additional training will be supplied before they start data collection. The study design presented here will provide the first robust evidence of the impact of cash transfers on the incidence of malnutrition in an emergency context with high levels of insecurity.

### Study status

The malnutrition risk factors surveys have been completed and preliminary analysis undertaken. The surveillance system data collection is ongoing and it is expected to be completed in June, 2017. Collection of process evaluation data is ongoing and qualitative data collection started in March 2017.

## Abbreviations

ACF: Action Againts Hunger
CHW: Community Health Workers
CTP: Cash Transfer Programmes
FSNAU: Food Security and Nutrition Analysis Unit
GAM: Global Acute Malnutrition
IDDS: Individual Dietary Diversity Score
IDP: Internally Displaced Persons
MCHN: Maternal and Child Health and Nutrition
MUAC: Mid Upper Arm Circumference
MEB: Minumum Expenditure BasketNFI Non-food items
NFI: Non-food items
OTP: Outpatient Therapeutic Programmes
REFANI: Research on Food Assistance for Nutritional Impact
UCL: University College London
